# Development and validation of standard and real patient gallstone library using Fourier transform infra-red spectroscopy

**DOI:** 10.1186/s12876-022-02227-8

**Published:** 2022-03-28

**Authors:** Lena Jafri, Muhammad Abbas Abid, Humera Asif, Bilal Hashmi, Hafsa Majid, Farooq Ghani, Sibtain Ahmed, Imran Siddiqui, Aysha Habib Khan

**Affiliations:** Section Head Chemical Pathology, Department of Pathology & Laboratory Medicine, Aga Khan University, Karachi, Pakistan

**Keywords:** galLstone, FTIR, Library, Fourier transform infra-red spectroscopy, Spectroscopy

## Abstract

**Background:**

Analysis of the constituents of gallstones using various spectroscopic techniques assists in identification of the pathogenesis of gallstones. In the current study, using Fourier Transform Infra-Red (FTIR) Spectroscopy, a Gallstone Standard Library (GSL) and a Gallstone Real Patients’ Library (GRPL) were developed and validated for gallstone composition analysis.

**Methods:**

The study was conducted at the Department of Pathology & Laboratory Medicine, Aga Khan University, Pakistan. Pure standards (cholesterol, calcium carbonate, bilirubin and bile salts) and gallstone specimens were analyzed using FTIR Nicolet iS-5 Spectrometer from Thermo Fisher Scientific, USA. Thermo Scientific™ QCheck™ algorithm, embedded within the OMNIC™ software, was used to identify the unique spectral fingerprint of the patient samples to match with known, standard material. Matching of > 75% was considered acceptable. Validation for accuracy of the library was performed for twenty analyzed gallstones at an international reference lab.

**Results:**

Concerted search analysis was performed against the developed GSL consisting of 71 “pure component” spectrum divided into 5 types to generate the library. For the Gallstone Real Patient Library (GRPL), 117 patient samples were analyzed. Ninety-eight gall stones (83.8%) out of 117 stones matched with the developed GSL. Majority stones were mixed stones (95.92%), with cholesterol being the primary component (91.83%). Results of the developed library were 100% in agreement with the reports received from the external reference lab.

**Conclusions:**

The library developed displayed good consistency and can be used for detection of gallstone composition in Pakistan and replace the traditional labor- and time-intensive chemical method of gallstone analysis.

## Background

Gallstone disease is one of the most common presentation in gastrointestinal practice and a common etiology of abdominal pain and emergency surgery [1]. Gallstone presents in 10–20% of the population in the Western countries, and about a quarter of these patients eventually require the removal of their gallbladder due to the severity of symptoms [2]. In Pakistan, the reported prevalence of gallstones is 10.2% [3]. The factors that promote gallstone formation in Pakistani population include the genetic makeup and diet consumed in this part of the world [4]. Literature suggests that factors favoring stone formation include age, obesity, female gender, multiparity and high blood triglyceride levels [5]. Recent literature also points, acute changes in weight, intake of certain drugs, and a lack of physical exercise as factors in the development of symptomatic disease [6]. It is imperative to learn the composition of gallstones as it provides an insight to practitioners regarding the underlying etiology of the disease and aid to reach the decision of medical or surgical treatment [7, 8].

In Pakistan, the diagnostic facilities for assessing the composition of gallstones are primitive and the methods used are often outdated and defective. Traditionally, the most common method used to analyze the composition of gallstones was the chemical method [1]. This method, although largely used previously, has multiple drawbacks such as high consumption of time and labor, and an incomplete analysis of the true composition of the gallstones. Inaccuracy in results is a major drawback [1]. Fourier transform infrared (FTIR) spectroscopy is the method used for quick, cost-effective and accurate analysis of gallstones [9]. The method analyzes the composition of the stone, and the results are compared to an already developed and validated library for confirmation and categorization. FTIR has been the tool of choice for classifying a large number of gallstone samples. It is applicable to all types of gallstones, and requires a small amount of sample without any pre-treatment [9]. Channa et al. performed a study in Pakistan analyzing gallstones using FTIR for their constituents including cholesterol, calcium carbonate, bilirubin and calcium bilirubinate [8]. No gallstone library was defined which can be used for large-scale gallstone sample analysis at other institutions. Since the FTIR technique provides fast and accurate results, a need was felt to develop and validate a gallstone standard and real patients’ library.

The aim of the current study was to develop and validate a Gallstone Standard Library (GSL) and a Gallstones Real Patient Library (GRPL) for the analysis of gallstones’ compositions for the FTIR method with a zinc selenide attenuated total reflection accessory (ATR).

## Materials and methods

The study was conducted at the Section of Chemical Pathology, Department of Pathology & Laboratory Medicine, Aga Khan University, Karachi, Pakistan. Exemption was obtained from the Aga Khan University’s Ethics Review Committee (2020–5796–15110). The study was conducted in three phases as described below.

### Phase I: preparation of standards & development of GSL

Samples were analyzed using FTIR Nicolet iS-5 Spectrometer with a Zinc Selenide ATR accessory from Thermo Fisher Scientific, USA. For each standard run, spectra were studied and wavelengths were noted for complete spectrum in the frequency range 400–4000 cm^−1^ at 4 cm^−1^ resolutions. The ATR head was twisted to one-click to apply the optimum pressure on the sample by the tip, as specified by the Instructions Manual of the device. A blank background spectrum was collected before each analysis to attain a relative scale for absorption intensity and blank subtraction. This ensured that all results were exclusively due to gallstone samples with no background noise. Each sample was run 16 times for optimization of the produced spectrum. After each analysis, the Zinc Selenide crystal and plate were wiped clean using 70% isopropanol.

Standards of cholesterol (C_27_H_46_O), calcium chloride anhydrous (CaCl_2_), bilirubin 99% and bile salt from Sigma-Aldrich Corporation, USA, were used for developing the GSL. The complete spectra of all four standards and their different combinations were saved as “Gallstone Standard Library.” Each standard was run twenty times on the FTIR for precision measurement of the standard spectra with calculation of mean, SD and CV for each spectrum. The resulting chromatograms were saved in the computer in the instrument-specific OMNIC™ software on the computer.

### Phase II: development of GRPL & generation of reports

To develop GRPL, gallstone samples received from May 2015 to November 2020 were saved in the clinical laboratory in dry ambient conditions. These stones were air-dried and stored at room temperature. Morphological features of the stones, such as color, shape, size, weight and number of stones was noted. Subsequently, stones were powdered using pestle and mortar before FTIR analysis. Spectra developed were matched with standards already saved in the computer as GSL and saved in “Gallstones Real Patients Library.” In each case, visual inspection of spectra matching was also performed to check the results by a technologist and a pathologist, and spectra were saved in GRPL. Thermo Scientific™ QCheck™ algorithm, embedded within the OMNIC™ software, was used to identify the unique spectral fingerprint of the patient samples to match with known, standard material. Matching of > 75% was taken as acceptable.

### Phase III: validation of GRPL

For validation of the GRPL, 20 gallstone specimens of real patients analyzed at AKU and spectra compared against the GRPL were sent to the College of American Pathologists (CAP) accredited international lab (Mayo Clinic Lab, Rochester, USA). This data was used to check for the accuracy of the GRPL library.

### Statistical analysis

The Statistical Package for the Social Sciences (SPSS) version 23 and Evaluation Protocol (EP) Evaluator were used for data analysis. Frequency of gender, weight, size and composition of stones were generated along with mean and standard deviation (SD) for quantitative variables. Mean, SD and CV were computed to assess precision of spectra. Concordance between standard spectrum and patient samples was calculated. Cohen’s Kappa was used to calculate concordance for external validation.

## Results

### Characteristics of gallstone spectra developed in phase I

**Fig. 1:**
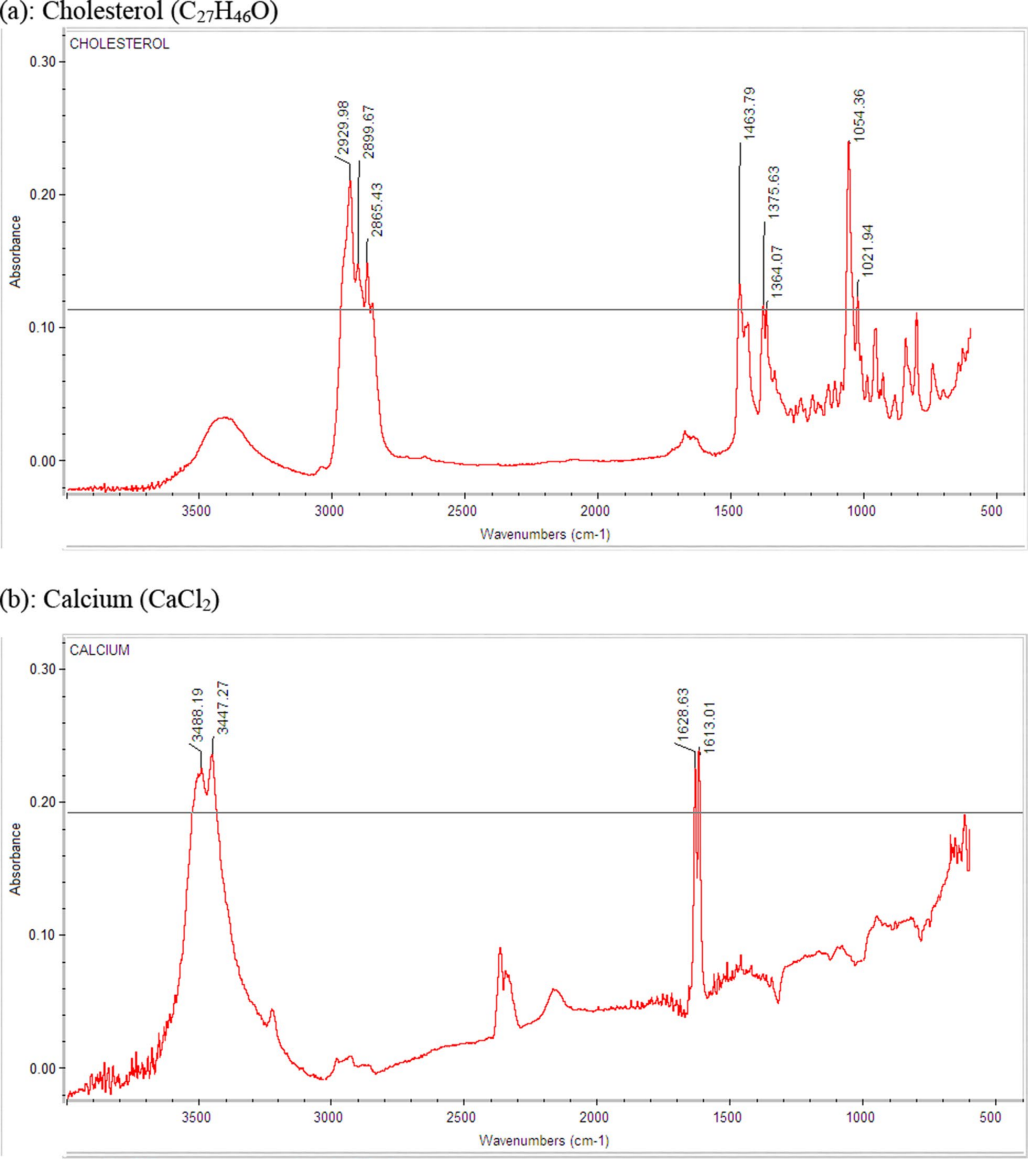

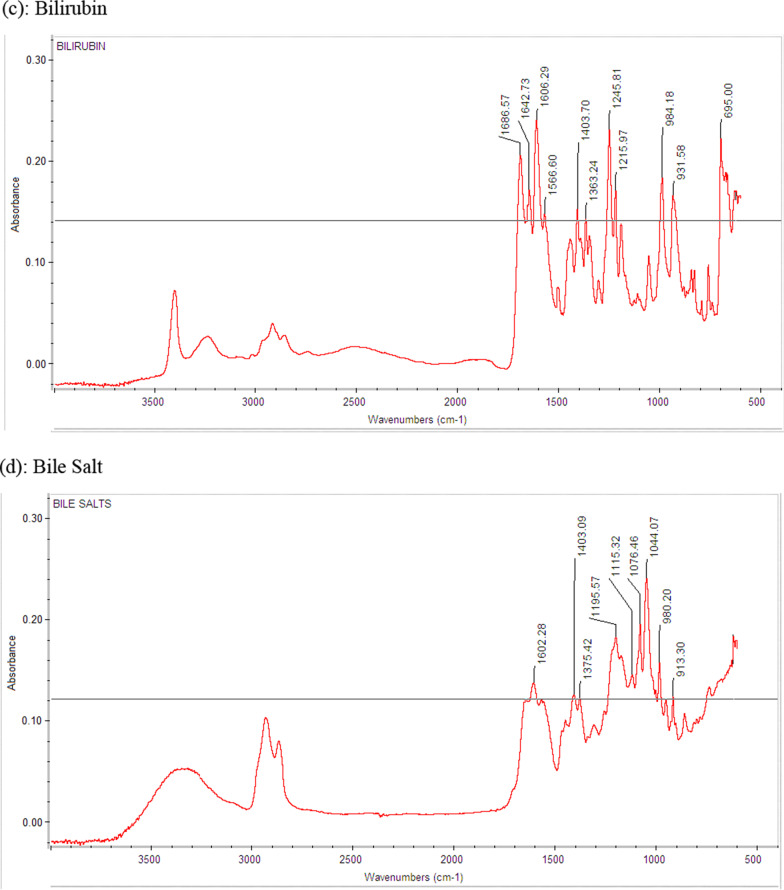
IR spectra of pure standards cholesterol, calcium, bilirubin, bile salt (**a**–**d**) displaying typical IR peaks by FTIR (Fourier Transform Infra-Red) Spectroscopy

Total seventy-one spectra were generated after analyzing pure standards (n = 30) and combination of these standards (n = 41). The pure standards in the GSL were labelled as cholesterol, bile salt, calcium carbonate, bilirubinate, and mixed. Mixed stones were further divided into 2 subtypes, based on the predominant constituent. Figure 1a–d show the typical FTIR spectra of pure cholesterol, bile salt, bilirubin and calcium standards respectively. All standards were characterized by the IR bands between 695 cm^−1^ to 3490 cm^−1^. The means of Infra-Red (IR) Bands/peaks produced by the standards are described in Table 1.

**Table 1:** Precision Study of 31 peaks of the four pure standards in gallstone analysis

Pure standards	Mean IR bands observed (cm^−1^)	SD	CV (%)
Cholesterol	2930.4	0.27	0.0
	2899.9	0.19	0.0
	2865.7	0.15	0.0
	1464.2	0.18	0.0
	1375.8	0.17	0.0
	1364.4	0.20	0.0
	1054.8	0.21	0.0
	1022.4	0.14	0.0
Calcium	3487.2	3.15	0.1
	3449.3	3.8	0.1
	1628.0	0.96	0.1
	1613.2	0.67	0.0
Bilirubin	1687.2	0.30	0.0
	1643.7	0.40	0.0
	1607.0	0.21	0.0
	1567.3	0.17	0.0
	1404.4	0.21	0.0
	1363.6	0.05	0.0
	1246.6	0.26	0.0
	1216.6	0.13	0.0
	984.8	0.19	0.0
	932.5	0.16	0.0
	695.7	0.09	0.0
Bile Salt	1601.7	0.84	0.1
	1403.0	1.14	0.1
	1375.3	0.40	0.0
	1196.0	0.29	0.0
	1076.8	0.12	0.0
	1044.1	0.37	0.0
	980.6	0.19	0.0
	913.7	0.15	0.0

### Characteristics of gallstone spectra from GRPL

Gallstone specimens from 117 patients were received in five years’ period from across Pakistan where majority were from females (n = 88; 77.2%). Mean age of the subjects was 45.4 ± 15.4 years. On physical examination of the gallstones, majority of the gall stones were green in color (52.6%) followed by brown (17.9%), yellow (11.5%), greenish brown (7.7%), black (5.1%), grey (3.8%) and white (1.2%). The mean weight of the stones was 3.89 ± 4.9 gm and their mean size was 1.14 ± 0.64 cm.

Unique spectral fingerprint for all 117 gallstones were produced. Concerted search analysis was performed against the developed GSL consisting of 71 “pure component” spectrum. Out of 117 samples studied 98 gall stones (83.8%) matched with the GSL and saved as GRPL. Majority stones were mixed stones (95.92%), with cholesterol being the primary component (91.83%). The description of these ninety-eight stones, along with the means of (IR) Bands/peaks produced by the patient specimens are listed in Table 2.

**Table 2:** Composition and the unique IR peaks observed in the 98 matched specimens

Stone type	Number of stones n (%)	IR peaks observed
Cholesterol only	4 (4.08%)	1022, 1051, 1366, 1463, 2865, 2899, 2934
Cholesterol + Bile Salt + Bilirubinate	90 (91.83%)	1022, 1051, 1366, 1463, 2865, 2899, 2934, 984, 1049, 699, 984, 1249, 1366, 1572
Bile Salt + Calcium carbonate + Bilirubinate	4 (4.08%)	984, 1049, 1628, 3445, 699, 984, 1249, 1366, 1572

### Validation of the GRPL with international lab

The results of the developed library were in 100% concordance with the reports received from an international CAP-accredited reference lab (Mayo Clinic Lab). However, only the major constituent of the stone (cholesterol; n = 20) was reported by the reference lab. Minimal amount of bile salts and bilirubin were also identified in forty percent (n = 8) of our analysis at AKU, as described in Table 3.

**Table 3:** Comparison of predominant constituents identified in gall stones by FTIR AKU with international reference laboratory

	Inter-laboratory comparison, n = 20		Concordance (%)
	Reference lab	AKU	
Main constituent of stone	Cholesterol	Cholesterol	100
Additional constituents identified in gall stones	None	None, n = 11 Bile salt & Bilirubin, n = 8 Only Bilirubin, n = 1	55

## Discussion

In the current study, a gallstone library for identification and analysis of gallstone samples by FTIR using the zinc selenide ATR accessory was developed and validated. FTIR allows for quick analysis of gallstones [1]. The ATR accessory enables the analysis of neat stone material without any pretreatment and without exposure to hazardous chemicals [10]. This makes the analysis less time- and labor-intensive [10]. The results generated using FTIR methodology are more comprehensive and accurate as compared to the traditionally used semi-quantitative titrimetric-colorimetric method [10].

Knowledge of the composition of gallstones is essential for the etiopathogenesis of the disease [11]. Abnormalities responsible for cholesterol gallstone formation include cholesterol supersaturation, defective conversion of cholesterol to bile acids or interruption of enterohepatic circulation of bile acids [12]. Pigment stones occur when red blood cells are being destroyed [13, 14]. Likewise, factors affecting gallstone formation include age, gender, parity, genetic makeup, obesity, body fat distribution, rapid weight loss, type of diet, physical activity, certain drugs and a history of diabetes [12]. Information about the composition of gallstone provides insight regarding the etiology of the stone and ongoing processes in the body.

The developed library was used to match real patient samples’ absorbance patterns against standard patterns. The results not only allowed for quicker and cheap analysis of gallstones; the results were also more detailed as compared to traditionally used chemical method. By far, the most common stone present in the study population was mixed stone, with cholesterol being the major constituent. Previous studies in the region also report cholesterol as the most common constituent. Channa et al. analyzed 109 gallstones in Jamshoro, Pakistan and reported cholesterol as the most common component of the gallstones [8]. They reported that pure cholesterol stone was much more common than cholesterol mixed with other constituents. This is in contrast to our study where majority of stones were primarily cholesterol mixed with other constituents. Khand et al. performed FTIR analysis on 77 gallstones from Jamshoro, Pakistan and also reported cholesterol (77.92%) as the most common constituent [15]. Samra et al. and Tahir et al. studied the composition of gallstones in patients in Multan, Pakistan and also reported cholesterol as the chief component in up to 82% of stones [16, 17]. These findings are in line with the results of our study where cholesterol was noted as the chief component. The IR peaks observed in this study and those in similar studies in the region are listed in Table 4.

**Table 4:** Comparison of IR peaks observed in standard and specimen compared with published literature

Analyte name	IR peaks in GSL	IR peaks in GRPL	IR peaks in Naseema et al. study [8]	IR peaks in Ha and biomaterials research [9]
Cholesterol	1022, 1055, 1364, 1376, 1464, 2866, 2900, 2930	1022, 1051, 1366, 1463, 2865, 2899, 2934	1054, 1463, 2865, 2899, 2929	1055, 1373, 1458, 2853, 2934, 3410
Bilirubin	696, 933, 985, 1217, 1247, 1364, 1404, 1567, 1607, 1644, 1687	699, 984, 1249, 1366, 1572	1246, 1607, 1683	1251, 1447, 1566, 1624, 1663, 3398
Calcium	1613, 1628, 3449, 3487	1628, 3445	854, 1338	855, 872, 1420, 1458, 1464, 1624,
Bile Salt	981, 1044, 1077, 1196, 1375, 1403, 1602	984, 1049	NA	NA

Though most of the IR peaks matched with those found in other studies from the region, samples of calcium presented unique IR bands compared to previous studies from the region. After careful rechecking of the standards and the real patient samples, it was concluded that peaks around 1628 and 3445 cm^−1^ were unique as none of the studies from this region reported these peaks. Ha et al. reported a similar peak at 1624 cm^−1^ while Hermida et al. reported an IR peak for Calcium at 3400 cm^−1^. [9, 18] Since these bands are unique to only our study in the region, cation will be exercised moving forward as calcium bands will be carefully examined to locate any erroneous results.

This pattern of composition is in contrast to the findings from sub-Saharan Africa where cholesterol was absent from most patient samples and China where bilirubinate was the most common constituent [19, 20]. This difference could possibly be due to a difference in diet, genetic makeup, or a combination of both. None of the studies performed in the region developed a library for use with gallstone specimen in future. This was a first known attempt of the development of a local library for large-scale analysis of gallstone at a major tertiary-care center in Pakistan.

While visually inspecting spectra matching, overlapping was noted in certain IR peaks. One peak was found to overlap between cholesterol and bilirubin (1364 cm^−1^), while one peak was found to overlap between bilirubin and bile salt (1403/1404 cm^−1^). This calls for good precision studies, and also requires careful interpretation by a pathologist before disseminating results.

There were limitations of the study. The real patient samples analyzed were limited in number. The samples were collected over a five-year period and additional samples will be serially examined and checked for the accuracy. Additionally, no prior commercially available or regional gallstone library was present to compare the results. There are intrinsic limitations of FTIR, as most gallstones are a mixture of several chemical substances and the absorption bands often overlap [9]. All of the studies performed in Pakistan analyzed gallstones on FTIR were for experimental use; none developed a library for widespread use in gallstone analysis. The developed GSL is a first known attempt to create a gallstone for use with FTIR in Pakistan. Not only will the library aid in the analysis of gallstones at one center; it will also serve as a national library for identifying the composition of gallstones in Pakistani population. Additional samples will be continuously added to the library for additional diversity, comprehensiveness and accuracy. Previously, Khan et al. used a 60% cutoff to declare a successful match for kidney stones. It was against a commercially available library [21]. Since we developed our own library for gallstone, a 75% cutoff was used for increased accuracy of analysis. The FTIR is one of the numerous techniques currently employed to analyze gallstones. Other techniques most commonly used include atomic absorption spectroscopy, energy‐dispersive X‐ray fluorescence, inductively coupled plasma atomic emission spectroscopy/mass spectrometry, laser‐based spectroscopic techniques (laser‐induced plasma spectroscopy/laser‐induced breakdown spectroscopy/laser ablation ICP‐MS), time‐of‐flight secondary ion mass spectroscopy, particle‐induced X‐ray emission, and scanning electron microscopy attached with energy‐dispersive X‐ray [22]. The next avenue of diagnostics and research would be to compare the results produced using FTIR spectrometry against other techniques and check for accuracy and precision of results produced.

## Conclusion

The developed GSL allows for quick, accurate and detailed results and can serve a nidus for a national library for used across the country. The library was successfully validated internally and externally. It paves the way for immediate upgradation of gallstone analysis to the FTIR technique and will also allow for national collaboration between clinical laboratories by disseminating and populating the library across the country.

## Data Availability

The datasets used and/or analysed during the current study are available from the corresponding author on reasonable request.

## Abbreviations

FTIR: Fourier transform infra-red
GSL: Gallstone standard library
GRPL: Gallstone real patients’ library
ATR: Attenuated total reflection accessory
CAP: College of American pathologists
SPSS: Statistical package for the social sciences
EP: Evaluation protocol
SD: Standard deviation
